# Consequences of a cosolvent on the structure and molecular dynamics of supramolecular polymers in water†
†Electronic supplementary information (ESI) available: Instrumentation, methods and supplementary figures. See DOI: 10.1039/c8sc02257g


**DOI:** 10.1039/c8sc02257g

**Published:** 2018-06-11

**Authors:** René P. M. Lafleur, Xianwen Lou, Giovanni M. Pavan, Anja R. A. Palmans, E. W. Meijer

**Affiliations:** a Institute for Complex Molecular Systems , Eindhoven University of Technology , P.O. Box 513, 5600 MB Eindhoven , The Netherlands . Email: e.w.meijer@tue.nl ; Tel: +31 040 2473101; b Department of Innovative Technologies , University of Applied Sciences and Arts of Southern Switzerland , Galleria 2, Via Cantonale 2c, CH-6928 Manno , Switzerland

## Abstract

Polar cosolvents are commonly used to guide the self-assembly of amphiphiles in water. Here we investigate the influence of the cosolvent acetonitrile (ACN) on the structure and dynamics of a supramolecular polymer in water, which is based on the well-known benzene-1,3,5-tricarboxamide motif. Hydrogen/deuterium exchange mass spectroscopy measurements show that a gradual increase in the amount of ACN results in a gradual increase in the exchange dynamics of the monomers. In contrast, the morphology of the supramolecular polymers remains unchanged up to 15% of ACN, but then an abrupt change occurs and spherical aggregates are formed. Remarkably, this abrupt change coincides with the formation of micro-heterogeneity in the water–ACN mixtures. The results illustrate that in order to completely characterize supramolecular polymers it is important to add time-resolved measurements that probe their dynamic behavior, to the conventional techniques that are used to assess the morphology of the polymers. Subsequently we have used time-resolved measurements to investigate the influence of the concentration of ACN on the polymerization and depolymerization rates of the supramolecular polymers. Polymerization occurs within minutes when molecularly dissolved monomers are injected from ACN into water and is independent of the fraction of ACN up to 15%. In the depolymerization experiments—initiated by mixing equilibrated supramolecular polymers with dissolved monomers—the equilibration of the system takes multiple hours and does depend on the fraction of ACN. Interestingly, the longest equilibration time of the polymers is observed at a critical solvent composition of around 15% ACN. The differences in the timescales detected in the polymerization and depolymerization experiments are likely correlated to the non-covalent interactions involved, namely the hydrophobic effect and hydrogen-bonding interactions. We attribute the observed fast kinetics in the polymerization reactions to the hydrophobic effect, whereas the formation of intermolecular hydrogen bonds is the retarding factor in the equilibration of the polymers in the depolymerization experiments. Molecular dynamics simulations show that the latter is a likely explanation because ACN interferes with the hydrogen bonds and loosens the internal structure of the polymers. Our results highlight the importance of the solution conditions during the non-covalent synthesis of supramolecular polymers, as well as after equilibration of the polymers.

## Abstract 2

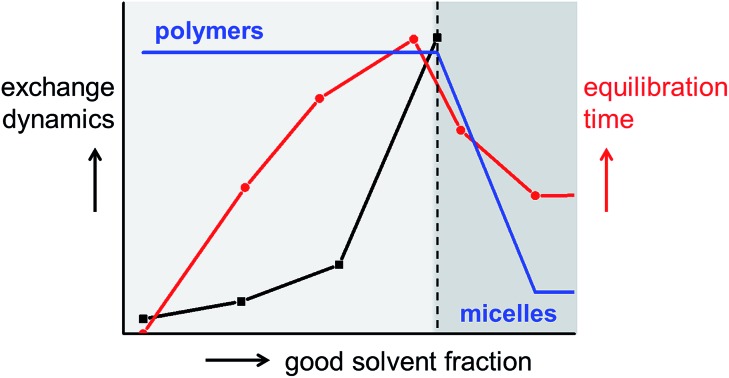
A cosolvent that is used to guide the self-assembly of amphiphiles in water causes abrupt structural changes, as well as non-linear behavior in the molecular dynamics of the amphiphiles.

## Introduction

In recent years small molecules have been synthesized that self-assemble in water to form one-dimensional supramolecular polymers and biomedical applications employing these polymers have started to emerge.^1^–^3^ The non-covalent interactions between the monomers is one of the key features of supramolecular polymers giving rise to interesting properties such as stimuli-responsiveness and environmental adaptation.^4^ As a consequence, supramolecular polymers are dynamic entities in which monomers can associate or dissociate at any given point in time. The extent and rate to which monomers are able to move within or between supramolecular polymers determines the ability of the polymers, or the materials formed thereof, to adapt to changes in their environment. Future development of these polymers for biomedical applications will therefore rely on the characterization of, and ultimately gaining control over, the kinetic properties of supramolecular polymers in water.

The majority of small molecules that are prepared with the aim of forming supramolecular polymers in water are amphiphilic, *i.e.* they contain a hydrophobic part as well as a hydrophilic part.^3^ Typically, these amphiphiles cannot be directly combined with water to form supramolecular polymers. The monomers are often first dissolved in a good solvent and are subsequently transferred to water, sometimes with removal of the cosolvent but not always. This methodology is not limited to small supramolecular building blocks,^5^,^6^ it is also a well-established method to assemble block copolymers in water,^7^–^10^ and central to inducing the light emission of aggregation-induced emission fluorogens.^11^–^13^ The solution conditions that are temporarily encountered during such a non-covalent synthesis protocol will have a profound influence on the supramolecular architectures that are formed.^14^–^16^ To control a non-covalent synthesis in water towards the target architectures, it is therefore important to consider the consequences of the cosolvent on the kinetics of supramolecular processes.

The solvent composition has been shown to influence the architecture of supramolecular nanostructures in equilibrated solutions.^17^–^22^ In addition, experimental studies have highlighted the time-dependence of these morphological transformations, using for example perylene diimides.^15^,^23^,^24^ The cosolvent dependent time evolution of these transformations was studied with spectroscopic measurements such as UV-visible absorption and circular dichroism, and recently also with luminescence when using amphiphilic platinum(ii) complexes.^25^ Kinetic and coarse-grained models were developed and qualitatively capture the experimental observations.^26^,^27^

Few groups have observed a correlation between the morphology of supramolecular aggregates and the thermodynamics of the solvent mixture.^18^,^28^,^29^ Whereas a solvent mixture may appear homogeneously mixed at a macroscopic length scale, on a nanometer length scale one component may self-aggregate resulting in altered interactions between the solvent and the supramolecular aggregates studied.^20^,^30^ For example, the morphological transitions of a discotic amphiphile were observed to be intimately connected to the enthalpy of mixing water and isopropanol.^18^ Likewise, an alcohol in organic solvents can interfere with supramolecular polymerization depending on whether the temperature allows the alcohol to self-aggregate into hydrogen-bonded clusters or not.^28^ In water, acetonitrile (ACN) molecules can cluster at a nanometer length scale without phase separation. It is suggested that up to about 20% ACN in water, the ACN molecules fill open space in the water structure. In the range of roughly 20–70% ACN in water, the solvent molecules self-associate and the mixture has coexisting clusters of water-rich and ACN-rich domains.^31^,^32^ This self-aggregation of a solvent may also influence the kinetic behavior of supramolecular polymers.

Kinetic studies on morphologically equilibrated supramolecular aggregates formed from *e.g.* peptide amphiphiles and discotic molecules were performed in the absence of a good solvent, or in the presence of marginal amounts thereof. These studies focused either on the internal dynamics of the aggregates,^33^ or the exchange of molecules between the supramolecular aggregates.^34^–^37^ Recently, to circumvent the influence of attached probes like fluorescent dyes on the molecular movements, we introduced the hydrogen/deuterium exchange mass spectrometry (HDX-MS) method as a novel alternative for supramolecular polymers.^38^ This method is well-known for studying proteins and was applied to a series of amphiphilic monomers that contain the benzene-1,3,5-tricarboxamide (BTA) motif within their hydrophobic pocket. These monomers self-assemble to form supramolecular polymers with a large aspect ratio.^39^,^40^ The polymers were diluted into D_2_O and the exchange of the amide hydrogen atoms for deuterium was followed over time to reveal the kinetics of all molecules present in solution.

Here, we have selected one BTA derivative as a model compound and investigate the influence of ACN as the cosolvent on the exchange dynamics of the polymers, as well as the supramolecular kinetics of their polymerization and depolymerization. A range of techniques is first used to analyze the influence of the solvent composition on the structure and kinetics of the supramolecular aggregates in equilibrium. Interestingly, we observe with HDX-MS measurements that even when there are no changes in the morphology, the cosolvent can have a significant influence on the exchange dynamics of the monomers. Supramolecular polymers in water are often processed by changing the solvent composition. Therefore, we study the kinetic response of our supramolecular polymers to two different mixing protocols that result in polymerization and depolymerization. Even though the final solution conditions are the same, we reveal that the method of preparation has an intriguingly large influence on the observed dynamics.

## Results and discussion

### Influence of solvent composition on the morphology

**Fig. 1:**
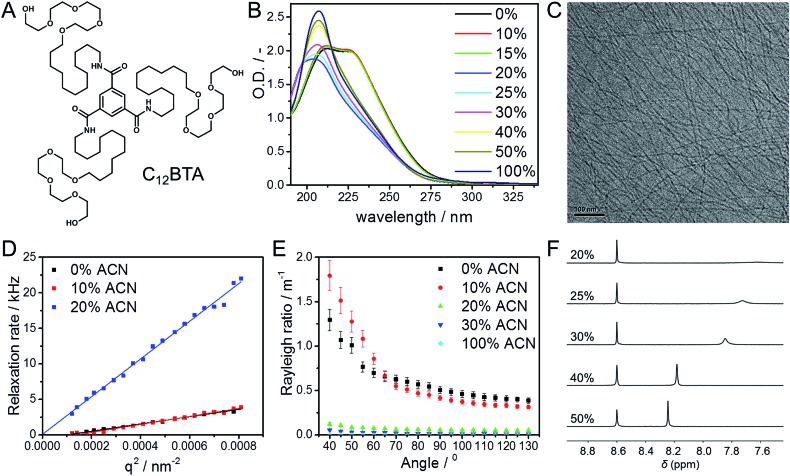
(A) Chemical structure of C_12_BTA. (B) UV-absorption spectra at several fractions of ACN in water (*T* = 20 °C, path length = 1 mm). (C) Cryo-TEM image in water containing 15% ACN, at a magnification of 25 000 and –10 μm defocus. (D) Relaxation rates as a function of *q*^2^ (*T* = 20 °C, path length = 1 cm). (E) Rayleigh ratio's as a function of the scattering angle (*T* = 20 °C, path length = 1 cm). (F) ^1^H-NMR spectra in D_2_O/CD_3_CN using pyrazine as internal standard at *δ* = 8.6 ppm. The concentration of C_12_BTA in all experiments was 582 μM.

The monomer used in this study is a *C*_3_-symmetrical BTA derivative; it contains a BTA core to which dodecyl aliphatic chains with four ethylene glycol units are attached (Fig. 1A, C_12_BTA). We studied the morphology of the self-assembled C_12_BTA in different solvent mixtures that contain various amounts of acetonitrile ACN (‘good’ solvent, in which the monomers dissolve) in water (‘poor’ solvent).

To this end, 582 μM C_12_BTA solutions in water were prepared using the heating–cooling protocol that was reported previously.^38^ Solutions of 582 μM C_12_BTA in ACN were prepared by adding ACN to the solid. The two solutions were combined in the appropriate ratio to obtain the desired volume fraction of ACN in water. The resulting mixtures were equilibrated at room temperature overnight. The morphology of the supramolecular aggregates was investigated at this concentration of 582 μM using a combination of UV-spectroscopy, cryogenic transmission electron microscopy (cryoTEM), light scattering, and ^1^H-NMR spectroscopy.

The UV-spectrum in pure water showed two absorption maxima at 212 nm and 223 nm, consistent with previous observations for an equilibrated C_12_BTA polymer in water (Fig. 1B, black line).^39^ The UV-spectra in the presence of 10% and 15% ACN were similar. In the presence of 20% ACN, a single broad peak centered at 203 nm was observed and the absorption was reduced. Upon further increasing the amount of acetonitrile, a hyperchromic effect was observed and the wavelength corresponding to the maximum absorption shifts to 207 nm in pure ACN. This wavelength corresponds to that of the molecularly dissolved C_12_BTA.^39^ These results indicate that the internal packing of the supramolecular aggregates differs at various fractions of good solvent, with limited changes up to 15% ACN. To exclude any influence of the preparation protocol on the spectral properties of the supramolecular aggregates,^15^,^41^ different preparation methods were employed and these led all to the same results (Fig. S1†).

The aggregates were subsequently visualized by cryoTEM. In the presence of up to 15% ACN, long fibers without ends, and a diameter of less than 10 nm, were observed (Fig. 1C). Despite multiple attempts at similar imaging conditions, visualization of aggregates formed in the presence of 20% ACN was not successful. This indicates that at ACN volume fractions above 15%, large aspect ratio supramolecular polymers are probably not present.

To complement the microscopy results, angular dependent light scattering measurements were performed. Analysis of the measured intensity autocorrelation functions of the samples containing 0%, 10%, and 20% ACN using the CONTIN method revealed that the data could only be fitted with two frequencies. However, since the contribution of one of the frequencies was typically small (<20%) as compared to the other one, only the largest amplitude modes were compared. The relaxation rates were all linearly proportional to the squared norm of the scattering vector (Fig. 1D). At 20% ACN, the linear relationship could be extrapolated through the origin, indicating that the observed relaxation rates correspond to translational diffusion. For the C_12_BTA samples in the presence of 0% and 10% ACN, the linear fit does not pass through the origin. Most probably, due to the long length of the polymers, dangling movements along the polymers also contribute to the relaxation rates. This is in line with the anisotropy in shape that was observed by cryoTEM.

To deepen our understanding, static light scattering experiments were performed from which the Rayleigh ratios could be calculated (ESI). An angular dependence of the Rayleigh ratio is observed for C_12_BTA aggregates in water and in the presence of 10% ACN (Fig. 1E). This pronounced angular dependence is lost at higher percentages of ACN. For all angles, the Rayleigh ratio at 0% and 10% ACN is larger as compared to the Rayleigh ratios at higher percentages of acetonitrile. The largest decrease in the Rayleigh ratio is observed when the percentage of ACN in the solution is increased from 10% to 20%. The Rayleigh ratios also decreased when increasing the fraction of good solvent to 30% or in pure acetonitrile (Fig. S2†), albeit to a lesser extent. The pronounced difference in the Rayleigh ratios between 10% and 20% ACN implies a structural transition of the aggregates. Combined with the results from dynamic light scattering, it can be concluded that this is a structural transition from large aspect ratio polymers to smaller, less-defined aggregates that can approximately be described as spherical aggregates.

Finally, ^1^H-NMR measurements were performed to investigate the influence of the solvent conditions on the aggregates formed from the C_12_BTA, in the corresponding deuterated solvents. ^1^H-NMR spectra in between 0% and 20% ACN were recorded to probe the structural transition from spherical aggregates to polymers. As expected, the aliphatic signals disappeared completely from the spectra below 20% ACN because of the presence of the polymers (Fig. S3†). Upon increasing the ACN fraction from 20% to 40%, the aromatic signals first appear as relatively broad peaks (Fig. 1F). At 40% and 50% ACN, the sharpness of the aromatic signals increased, and the signals shifted downfield towards the chemical shift usually observed for the molecularly dissolved C_12_BTA. Hence, the results from ^1^H-NMR also reveal a gradual disassembly of the spherical aggregates in response to an increasing fraction of ACN. This is consistent with the gradual decrease in the Rayleigh ratios at ACN fractions above 20%. The latter decrease was also relatively small as compared to the large change in the Rayleigh ratio that was attributed to an abrupt change in the aspect ratio of the aggregates.

To summarize the section on the influence of solvent composition on the morphology, all experiments presented above show that the C_12_BTA is self-assembled into large aspect ratio cylindrical aggregates at ACN fractions up to 15%, which we will refer to as supramolecular polymers. In between 15% and 20% ACN, there is an abrupt morphological change to smaller aggregates that are likely spherical. These spherical aggregates are likely not stabilized by hydrogen bonds and fully disassemble at ACN fractions above 40%. Remarkably, the critical solvent composition for the presence of supramolecular polymers correlates with the ACN fraction at which changes in the nanoscopic liquid structure of the binary solvent mixture occur.^31^,^32^

### Kinetics of supramolecular polymers under quasi-equilibrium conditions

The C_12_BTA contains six hydrogen atoms that will exchange for deuterium once the supramolecular aggregates are diluted into D_2_O.^38^ The peripheral alcohols are in contact with water and therefore exchange immediately. However, the amide hydrogen atoms that are contained in the hydrophobic pocket of the aggregates exchange at a slower rate, depending on solvent accessibility and hydrogen bonding.^42^ Therefore, kinetic HDX profiles can be constructed by following the conversion of the amides into their deuterated analogs.

Before starting the HDX-MS experiments in the presence of ACN, we investigated whether the dilution step causes changes in the aggregated state of the C_12_BTAs. Equilibrated samples at 582 μM C_12_BTA in the presence of ACN were diluted 50-fold or 100-fold into solutions with equal solvent composition. The typical shapes of the UV-absorption spectra with two absorption maxima <20% ACN, and one absorption maximum at 20% ACN, were observed also at low micromolar concentrations (Fig. S4†), indicating that the molecular packing of the C_12_BTAs does not change upon dilution.

**Fig. 2:**
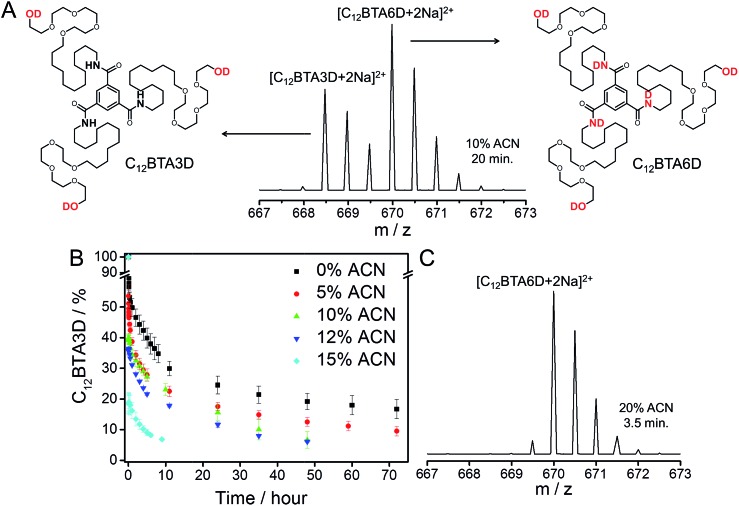
(A) Electrospray ionization MS spectrum of a HDX experiment that shows the part of the *m*/*z* spectrum containing the doubly charged ion. The spectrum was obtained 20 minutes after the 100-fold dilution of a 582 μM C_12_BTA solution containing 10% ACN and H_2_O, into 10% ACN and D_2_O. The left isotope pattern corresponds to the chemical structure of C_12_BTA3D and the right isotope pattern corresponds to the chemical structure of C_12_BTA6D. (B) Percentage of C_12_BTA3D at multiple time points after the 100-fold dilution of a 582 μM C_12_BTA solution in H_2_O/ACN, into D_2_O/ACN, in the presence of 0% (black squares), 5% (red circles), 10% (green up triangles), 12% (blue down triangles) and 15% ACN (cyan diamonds). The ACN fraction was equal before and after the dilution. The hypothetical data point (*t* = 0 min, C_12_BTA3D = 100%) reflects the immediate exchange of the alcohols upon dilution, and 7% C_12_BTA3D is around the lower limit we can reliably detect.^38^ The error bars represent one standard deviation of uncertainty obtained from three separate kinetic experiments. (C) Electrospray ionization MS spectrum of a HDX experiment that shows the part of the *m*/*z* spectrum containing the doubly charged ion. The spectrum was obtained 3.5 minutes after the dilution of a 582 μM C_12_BTA solution containing H_2_O with 20% ACN, 100-fold into D_2_O with 20% ACN. The isotope pattern corresponds to C_12_BTA6D, the peak at 669.5 is due to the H_2_O still present after diluting the sample.

Time-resolved HDX-MS experiments were performed on 582 μM C_12_BTA in H_2_O/ACN solutions containing 5%, 10%, 12%, and 15% ACN. After equilibration, these mixtures were diluted 100 times into the corresponding D_2_O/ACN solutions. Because of the sensitivity of the C_12_BTA aggregates to the exact solvent composition, the volume percentage of ACN was accurately controlled in each step (ESI). Starting within minutes after the dilution step, we monitored the molecular weights of the C_12_BTA monomers using electrospray ionization MS. Consistent with earlier work,^38^ the mass spectra were observed to contain two species, one of which is C_12_BTA3D with deuterated alcohols, and the other one corresponds to C_12_BTA6D resulting from HDX of both the alcohols and the three amides (Fig. 2A).

C_12_BTAs with less than three deuterium atoms were not detected in excess D_2_O, because of the very fast exchange of the hydrogen atoms of the alcohols for deuterium. Also C_12_BTAs with one or two deuterated amides (C_12_BTA4D and C_12_BTA5D) were not present in all of these kinetic experiments. To illustrate the cosolvent dependence of the conversion of C_12_BTA3D into C_12_BTA6D, Fig. 2B displays the percentage of C_12_BTA3D as a function of time. For comparison, we added the HDX profile recorded in pure water (black squares) that was first presented in ref. 38. The C_12_BTAs in a solution containing 20% ACN, in which no supramolecular polymers are formed, were fully deuterated at the time of the first measurement (Fig. 2C).

The HDX profiles in Fig. 2B show that the HDX is very fast in the first minutes after the dilution step. At the time of the first measurements, 3.5 minutes after dilution, around 30% of the C_12_BTAs were converted into C_12_BTA6D in the absence of ACN, whereas 80% of the C_12_BTAs were fully deuterated in the presence of 15% ACN. After half an hour, the HDX rate was slower for all investigated cosolvent fractions. During the first hours, several data points recorded for samples containing <15% ACN overlap. The HDX could be followed for two days in the presence of 10% and 12% ACN, and for over three days in the presence of 0% and 5% ACN. All of these observations indicate that the HDX rates gradually increase upon increasing the fraction of acetonitrile from 0% to 15%. The rate constants were calculated by fitting the data with exponentials, and a comparison of the values that were obtained supports this conclusion (Fig. S5†). Most probably, ACN molecules weaken the interactions between the C_12_BTAs; when the fraction of ACN increases, the monomers inside the polymers become more accessible for the solvent, and are likely less involved in hydrogen bonding.^40^,^43^ At 20% ACN, full deuteration occurs immediately, which is likely due to the presence of only spherical aggregates.

### Cosolvent-dependent polymerization kinetics

After elucidating the exchange dynamics of monomers under quasi-equilibrium conditions, we investigate the influence of cosolvent on the kinetics of the (de)polymerization of the C_12_BTA monomers. Polymerization was induced by the common methodology of transferring the monomers that are dissolved in the good solvent (acetonitrile) to the poor solvent (water). The dispersion of the concentrated C_12_BTA solutions into water was accurately controlled using a Berger-ball mixer.^44^ Because this mixer is embedded in a stopped-flow setup, the formation of the supramolecular polymers and spherical aggregates can be studied as a function of time by following changes in the UV-absorption.

**Fig. 3:**
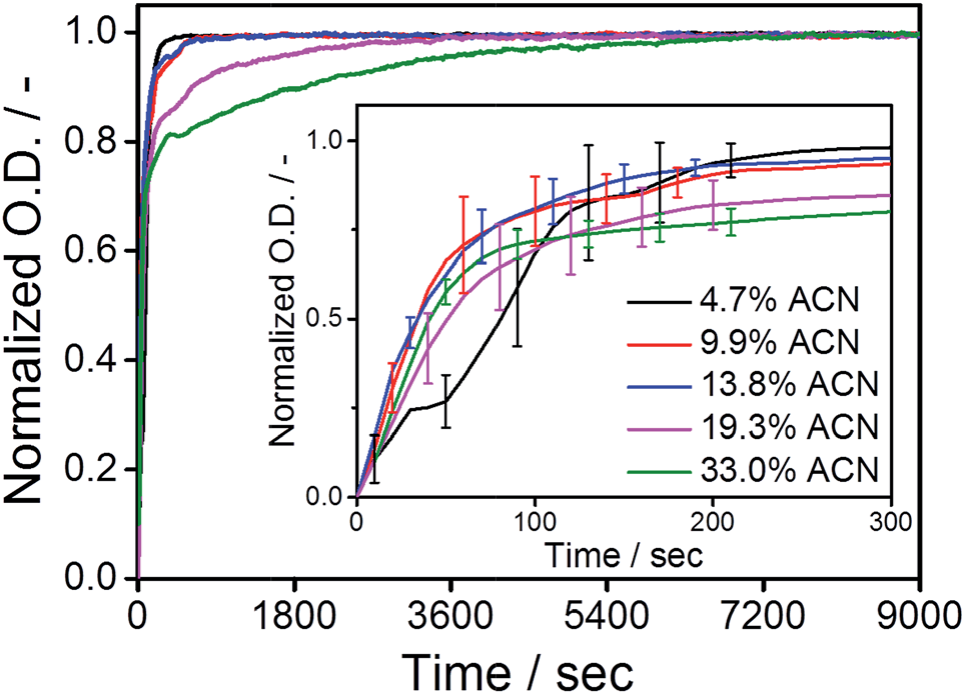
Normalized UV-absorption at 229 nm as a function of time, after mixing a concentrated solution of the C_12_BTA in ACN with pure water, for several fractions of ACN in the final mixture (*T* = 20 °C, path length = 1.5 mm). The red, black and blue lines correspond to the self-assembly of polymers. The purple and green lines correspond to the formation of spherical aggregates. The curves are an average of three measurements, and the inset shows a zoom of the first five minutes after mixing with the error bars representing one standard deviation of uncertainty.

Concentrated solutions of C_12_BTA were prepared in ACN, in which the monomers are molecularly dissolved. To induce their aggregation, the ACN solutions were, within milliseconds, efficiently mixed with pure water in a stopped-flow setup. All of the polymerization experiments were performed at a final C_12_BTA concentration of 582 μM. Subsequently, the UV-absorption at 229 nm was followed over time. This wavelength is close to the absorption maximum at 223 nm that is observed for the supramolecular polymers, and the absorption intensity at this wavelength is significantly lower when no supramolecular polymers are formed (Fig. 1B). The UV-absorption curves were normalized to facilitate a comparison of the timescales (Fig. 3). The full absorption spectra that were recorded 2.5 hours after mixing displayed the typical shapes with two absorption maxima in a solution containing <15% ACN, and one absorption maximum in a solution above 15% ACN (Fig. S6†). Interestingly, we observed that there are no significant differences in the association rates below 15% ACN. In these cases, the polymers self-assemble in approximately five minutes. In contrast, at larger fractions of ACN, spherical aggregates form and the UV-absorption of the aggregates was observed to equilibrate in one or two hours (purple and green lines for 19.3 and 33%, respectively).

### Cosolvent-dependent equilibration kinetics

The polymerization kinetics of the supramolecular polymers that form below 20% ACN were not affected by the different fractions of ACN. Therefore, we investigate whether the cosolvent affects the depolymerization kinetics of the polymers.

**Fig. 4:**
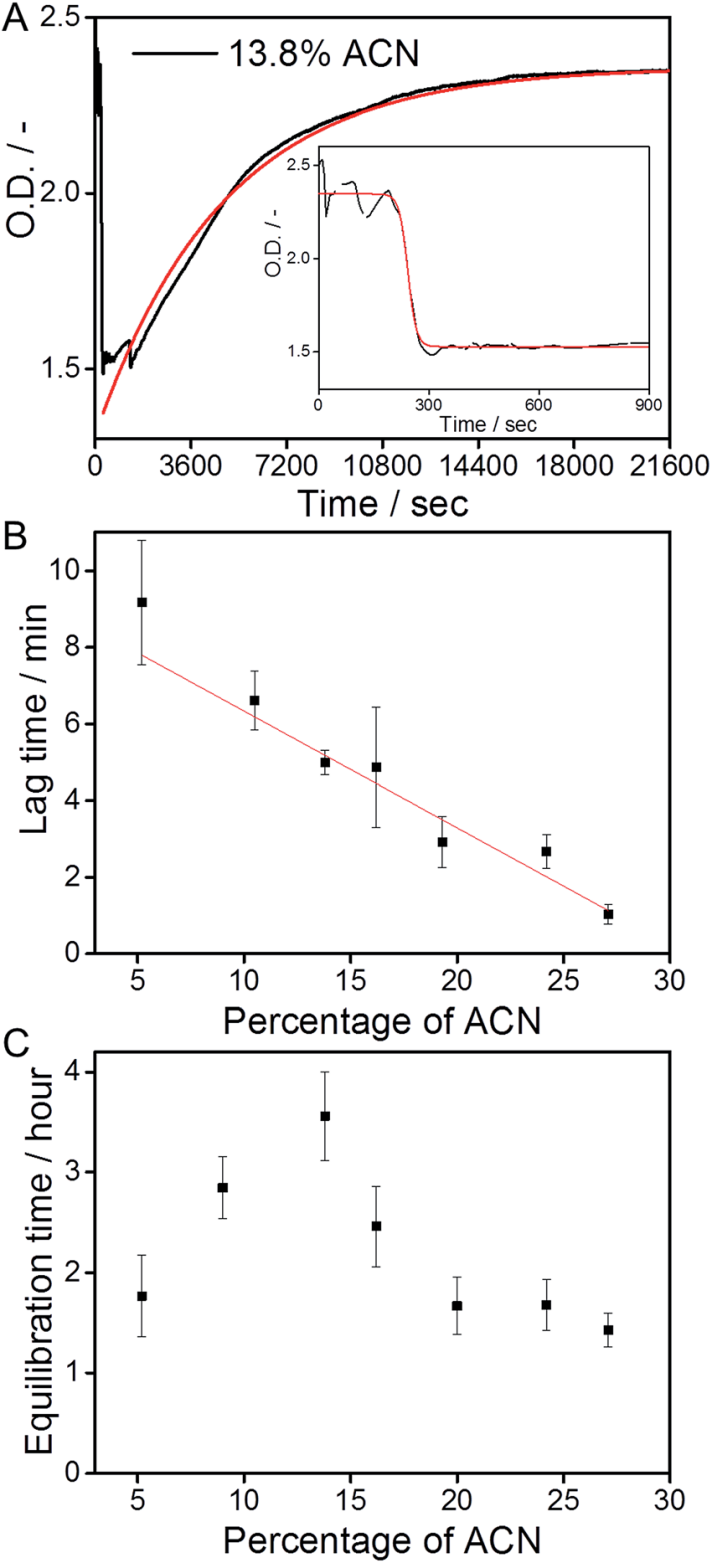
(A) UV-absorption at 229 nm as a function of time, after mixing two equally concentrated solutions of the C_12_BTA in ACN and the C_12_BTA in water, at a final ACN concentration of 13.8% (black line). Starting from the time point corresponding to the minimum UV-absorption value, a mono-exponential fit was applied (red line). The decrease in the first 15 minutes (inset) was fitted with a sigmoidal function (red line). (B) The average lag time (*t*-90 of the sigmoidal fits, black squares) computed from nine measurements, as a function of the ACN concentration. The straight line was obtained from linear regression. (C) The average equilibration time (*t*-90 of the exponential fits) computed from three measurements, as a function of the ACN concentration. All measurements were performed with a stopped-flow setup at a final C_12_BTA concentration of 582 μM (*T* = 20 °C, path length = 1.5 mm). All error bars represent one standard deviation of uncertainty.

The supramolecular polymers were forced into an out-of-equilibrium state by the mixing of equally concentrated solutions of the C_12_BTA in water and the C_12_BTA in ACN. Also this strategy was carefully controlled by using the Berger-ball mixer. Similar as for the other experiments, we performed the depolymerization experiments at a final C_12_BTA concentration of 582 μM. A typical kinetic UV-profile obtained by following the UV-absorption at 229 nm over time is displayed in Fig. 4A (black line). A few minutes after the mixing, a sudden decrease in the UV-absorption at 229 nm is observed (Fig. 4A, inset). After this fast decrease, a slow increase in the absorption is observed over multiple hours. The final UV-absorption value after equilibration depends on the morphology of the supramolecular aggregates formed in the solvent composition in question. However, the minimum UV-absorption value that is reached after the rapid decrease does not change significantly with the solvent composition (Fig. S7†). In control experiments with 0%, 3% and 5% ACN, no changes in the UV-absorption at 229 nm occurred (Fig. S8†). The latter observations suggest that the mixing process induces an initial common change in the molecular packing of the supramolecular polymers, provided that a sufficient amount of ACN is added during the mixing process. For that reason our first aim was to characterize the transient morphology of the supramolecular aggregates that is present after the rapid decrease of the UV-absorption.

**Fig. 5:**
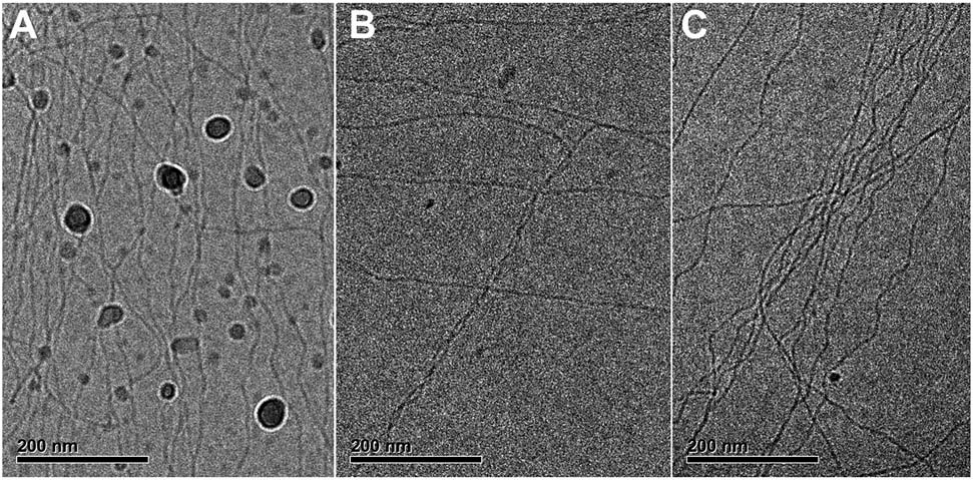
CryoTEM images of 582 μM C_12_BTA in 90% H_2_O + 10% ACN, at a magnification of 25 000 and –5 μm defocus. C_12_BTA samples in water and ACN were combined and subjected to (A) mixing at 10 000 rpm for 20 seconds with an Ultra-Turrax, (B) mixing at 10 000 rpm for 20 seconds with an Ultra-Turrax and waiting for 3.5 minutes, and (C) manual mixing by inverting the sample a couple of times, and waiting for 12 minutes. The samples were vitrified immediately after these procedures. The black sphere-like particles are crystalline ice particles.

We performed cryoTEM on C_12_BTA samples in water and 10% ACN directly after mixing. Two types of mixing were used to investigate whether the mixing protocol affects the morphology of the supramolecular polymers; manual mixing and high turbulence mixing using an Ultra-Turrax, which is representative for the Berger-ball mixer. The samples were vitrified at several time points and imaged. For all conditions, only long cylindrical aggregates, indicative of supramolecular polymers, were observed (Fig. 5). Hence, the morphologies of the supramolecular polymers formed from the C_12_BTA are stable. This suggests that the mixing process, either manually or controlled with a machine, causes only subtle differences in the molecular packing of the supramolecular polymers. Based on the HDX experiments (Fig. 2B) and polymerization kinetic results (Fig. 3), we anticipate that the association between the C_12_BTAs is relatively strong. When inspecting the UV-absorption spectra recorded at several time points after mixing the C_12_BTA solutions in water and ACN, we observe that the absorption peak centered around 223 nm decreases, resulting in a concomitant decrease in the absorption at 229 nm (Fig. S9†). This corroborates small changes in the packing of the C_12_BTA monomers, rather than a change in the morphology of the supramolecular polymer.

We next studied the intriguing kinetic phenomena as observed in Fig. 4A in more detail by investigating their cosolvent-dependence. Therefore we performed the same mixing experiments multiple times at various fractions of ACN in water, and quantified the different timescales involved. The rapid decrease in the UV-absorption was observed to consistently occur at a timescale of minutes after the mixing, and the decrease in absorption was fitted with a sigmoidal function. The slow increase in the UV-absorption values that was detected after the decrease, was fitted with a mono-exponential function (Fig. 4A, red lines). Both types of fits were used to calculate the time required to reach 90% of the absorption value (*t*-90) that was obtained after the decrease or increase, respectively. Because the solutions are not in equilibrium after the sudden decrease in the UV-absorption, the sigmoidal functions were applied to the first part of the UV-trace up to a time point at which the absorption did not show an apparent increase (for example: Fig. 4A, inset).

The *t*-90 of the initial decrease was defined as the lag time, because we observed that the abrupt decrease in the UV-absorption started at different time points depending on the concentration of ACN. After fitting the data we concluded that the lag time decreases when the percentage of ACN increases. The solvent-dependence of the lag time could be fitted with a linear equation (Fig. 4B). Most probably, this linear relationship in the kinetics is correlated with the polarity of the solvent mixtures, which also decreases linearly upon increasing the fraction of ACN.^31^ It is anticipated that when the solvent polarity of the mixture decreases, it becomes a better solvent for the more apolar part of the C_12_BTA monomers that constitute the hydrophobic core of the supramolecular polymers.^45^ In this case the solvent is more likely to interfere with the molecular packing of the polymers, and the linear trend as presented in Fig. 4B is proposed to be the kinetic manifestation of this effect.

The *t*-90s that were obtained from the mono-exponential fits that describe the slow increase in the UV-absorption (the equilibration of the supramolecular aggregates), reveal a distinctly different response to the solution conditions (Fig. 4C). The equilibration time increases up to 13.8% ACN, follows a decrease above 15%, and stabilizes when the contribution of ACN exceeds 20%. Hence, the longest equilibration time is observed close to the critical solvent composition of 15% ACN. In previous work, Korevaar *et al.* reported the same phenomenon for a one-dimensional polymer in organic solvents. It was explained with the competition between more or less equal rates of assembly and disassembly. Moreover the kinetic model indicated that cooperativity is a prerequisite for such kinetic behavior.^26^ Although recent results using coarse-grained simulations predicted the cooperative growth of polymers based on the C_12_BTA in water,^46^ this is the first experimental manifestation for a cooperative mechanism in this system. In the above we proposed that the solvent can interfere with the molecular packing of the monomers inside the supramolecular polymers. This proposal can also be used to rationalize the slowest equilibration at the critical solvent composition. The more ACN is present, the longer it will take to restore the molecular packing, *i.e.* the internal order, of the supramolecular polymers. Therefore, the equilibration time increases up to 15% ACN. Above 15% ACN, the C_12_BTA monomers will progress towards an equilibrium situation in which supramolecular polymers are no longer present. The depolymerization of the supramolecular polymers is increasingly favored when more of the good solvent is present, resulting in a shorter equilibration time.

Conceivably most striking is our finding that up to the critical solvent fraction, the polymerization rate of the supramolecular polymers is unaffected by the presence of the good solvent (Fig. 3), whereas its presence clearly influences the rates at which the molecular packing of the polymers will distort (Fig. 4B), and subsequently recover (Fig. 4C). Because the final solvent composition of the polymerization and depolymerization experiments is the same, these results indicate that the mixing protocol that is used to prepare the supramolecular polymers has a large influence on the kinetics.

The different kinetic observations can be explained by considering the driving forces for polymerization, which are the hydrophobic effect and hydrogen bonds between the monomers. Coarse-grained simulations indicated that the rate-limiting step for the formation of the polymers is the consolidation of the hydrophobic pocket by hydrogen bonds, since the initial hydrophobic collapse is very fast.^46^ The latter effect likely causes the fast rate at which the polymers assemble to be insensitive to small amounts of cosolvent (Fig. 3). However, the minute and hour timescales observed when mixing ACN with preformed polymers can be explained exclusively in terms of hydrogen bonding (Fig. 4). The active distortion of the molecular packing upon mixing is likely caused by solvent molecules that interfere with the hydrogen bonds between the C_12_BTAs, because the only long-range interaction that could account for the cooperative re-equilibration are hydrogen bonds.^47^,^48^

### Internal structure of supramolecular polymers in equilibrium

In order to provide a molecular rationale for the influence of ACN on the structure and dynamics of the supramolecular polymers, we performed all-atom molecular dynamics (MD) simulations. Starting from the equilibrated configuration of the atomistic supramolecular polymer that was obtained in our previous work,^45^ 10 mol% of the H_2_O molecules in the simulation box were substituted for ACN. Subsequently, the supramolecular polymer underwent an additional 250 ns of MD simulation in the ACN/H_2_O solution. During this MD simulation, ACN interacted with the polymer, eventually penetrating its hydrophobic pocket. It was also observed that ACN molecules locally interact with the cores of the C_12_BTA monomers, establishing intermittent hydrogen bonding with the amides of the C_12_BTAs (Fig. S10†).

**Fig. 6:**
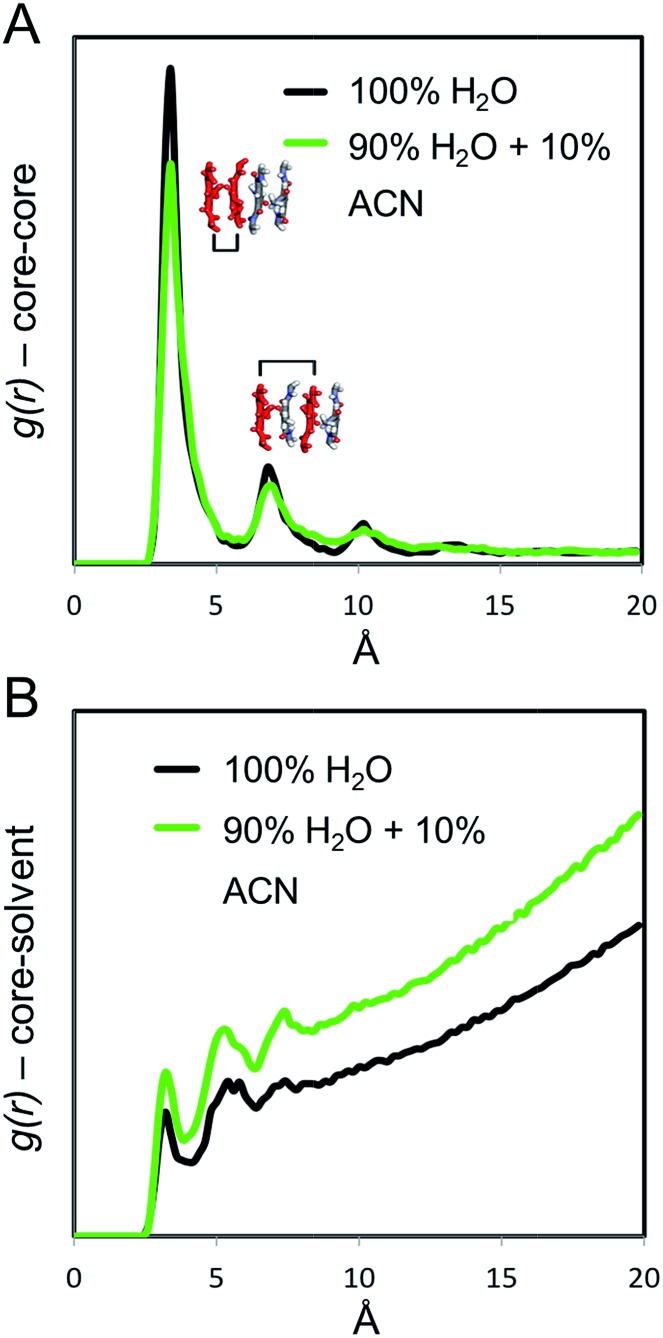
Radial distribution functions *g*(*r*) of the (A) C_12_BTA-cores, and of (B) C_12_BTA-cores–water. Both *g*(*r*) were calculated from the MD simulations in pure water (black) and in water with 10% ACN (green).

From the last 100 ns of the MD simulations in 100% water and in 90% water + 10% ACN, the radial distribution functions (*g*(*r*)) between the aromatic cores of the C_12_BTAs were computed. The *g*(*r*) provides information on the relative probability of finding C_12_BTA-cores in space as a function of the inter-C_12_BTA-core distance. The relative height of the *g*(*r*) peaks at ∼3.5 Å, and multiples thereof, are indicators of the stacking order/persistency in the supramolecular polymers (the higher the *g*(*r*) peaks, the more persistent is the core stacking).^43^,^45^,^46^ The maxima of the peaks in the presence of 10% ACN around 3.5 and 7 Å are lower as compared to pure water (Fig. 6A). On average, the core stacking of the C_12_BTAs is more persistent in pure water by ∼15% as compared to a solution containing 10% ACN. Subsequently, we calculated the core-water *g*(*r*) for the two different solution conditions. This *g*(*r*) indicates the relative probability of finding water molecules at a close distance from the C_12_BTA cores. Water penetration close to the C_12_BTA cores is more probable in 90% water + 10% ACN as compared to pure water (Fig. 6B).

The penetration of ACN has several important consequences. Firstly, it causes the polymer to swell on a sub-nanometer length scale. Secondly, it locally disturbs/destabilizes the hydrogen bonding between the C_12_BTAs because it favors the interaction with the amides of the C_12_BTAs. In addition, the water molecules also interfere with hydrogen bonds between the C_12_BTAs, augmenting the level of disorder in the polymer. The UV-absorption profiles obtained from the depolymerization experiments (*vide supra*) led us to propose that ACN interferes with the molecular packing of the supramolecular polymers. The MD simulations show that this is indeed a likely explanation.

Previous MD simulations clearly showed that the supramolecular polymers self-assembled from C_12_BTA are not extended in water, but strong hydrophobic effects induce folding of the polymers.^43^,^45^,^46^,^49^ In this way, the core of the polymers appears to be discontinuous; all along the polymers there are spots where the aromatic cores of the C_12_BTAs are more accessible/exposed to the solvent. Recent simulations demonstrated that these discontinuities along the stack of the C_12_BTA monomers are ‘exchange hot spots’ in the supramolecular polymers – points along the polymers where the monomers are more likely to leave the polymer, as compared to the average.^49^

**Fig. 7:**
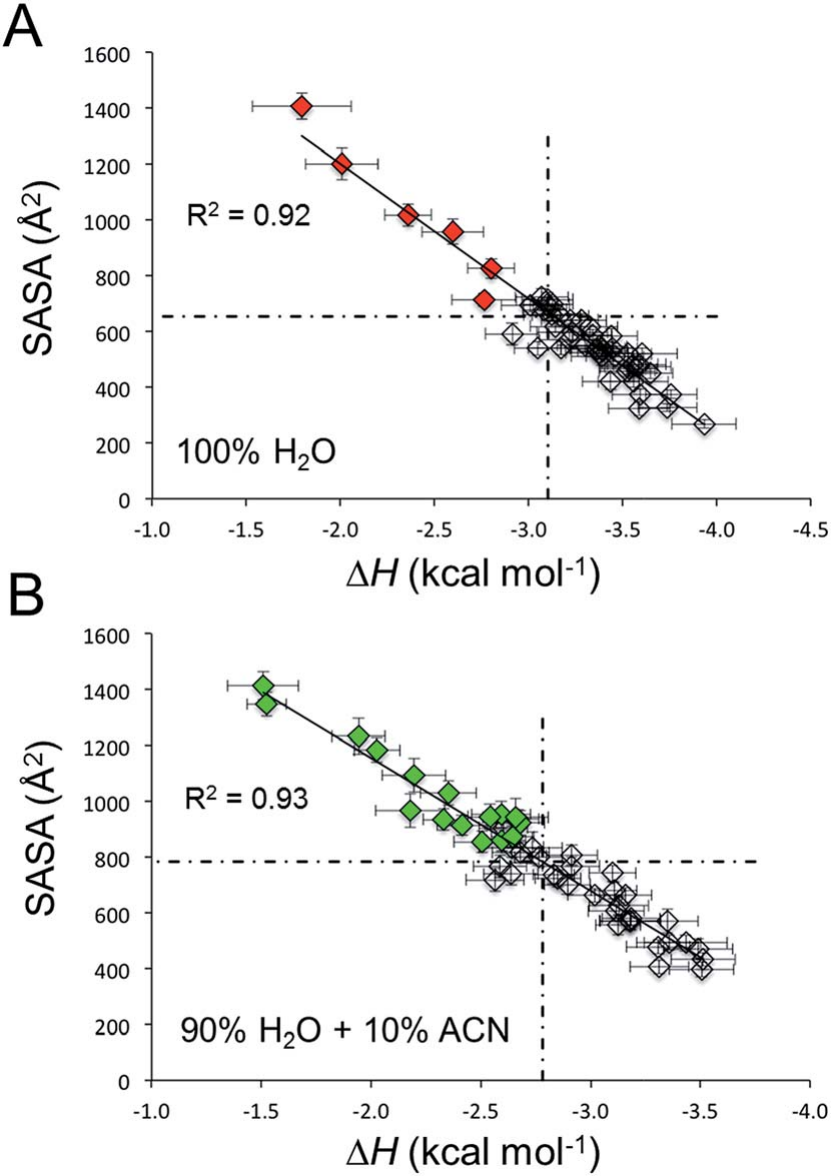
Exchange hot spots in the polymer in different solvents. The solvent accessible surface area (SASA) as a function of the pairwise incorporation energy (Δ*H*) in (A) pure water, and (B) in 90% water with 10% ACN. The dashed lines represent the average values of the monomers. Colored C_12_BTAs with a SASA and Δ*H* above/below the average, respectively, are exchange hot spots in the polymer. The error bars represent one standard error of the mean, *R*^2^ values represent the similarity to the linear trends obtained from linear regression.

In this perspective, for every C_12_BTA in these atomistic polymer models (composed of 48 C_12_BTAs), we calculated from the MD runs the solvent accessible surface area (SASA(*n*)) and the energy of incorporation (pairwise solute–solute + solute–solvent interaction: Δ*H*(*n*)) of each monomer in the C_12_BTA polymers. A comparison of the individual monomer values to the average calculated over all monomers indicates how much each monomer is exposed to the solvent, or more/less stably incorporated into the polymer. We observe the characteristic inverse linear relationship (Fig. 7A) seen also in our the previous studies.^49^ Those monomers more exposed to water, are also less stably incorporated into the polymer. These monomers can be more easily exchanged with the surrounding (exchange hot spots). We could clearly observe that immersing the same supramolecular polymer in 90% H_2_O and 10% ACN augments the average SASA of the monomers (from ∼650 Å^2^ in pure water to ∼800 Å^2^ in water with 10% ACN), and diminishes the strength of monomer incorporation into the polymer (from ∼–3.1 kcal mol^–1^ in pure water to ∼–2.8 kcal mol^–1^ in water with 10% ACN). Thus, the presence of ACN makes the monomers on average more exposed to the solvent and less stably incorporated into the polymer. These data well correlate with the swelling of the polymer and the interference with the hydrogen bonds as observed above. Even more interesting, this analysis shows that the number of exchange hot spots along the polymer grows in 10% ACN (Fig. 7B: green points with SASA and Δ*H* above and below the average, respectively), as compared to pure water (Fig. 7A: red points).

The kinetic HDX profiles as discussed above showed an increased rate of deuteration of the supramolecular polymers when the fraction of ACN is increased. The MD simulations are therefore remarkably useful to understand how these experimental observations could emerge from structural distortions at the length scale of a few nanometers. In 90% water + 10% ACN, the supramolecular interactions between the monomers are less persistent and this produces an increased number of exchange hot spots along the simulated polymer. In other words, the simulations indicate that more C_12_BTAs are incorporated less secure into the polymer and are therefore more likely to be released from the polymer. Although the experimental HDX results represent the average of all C_12_BTA molecules in the solution, the faster release of monomers from the polymers is likely to contribute to the faster deuteration of the amides. In this sense, the MD simulations provide a molecular-scale rationale that corroborates the results obtained with HDX-MS.

## Conclusions

Our kinetic experiments show that the use of a cosolvent can have a significant impact on the exchange dynamics of the monomeric units in supramolecular polymers in water. Despite this change in exchange dynamics, the morphology of the polymers persists up to a critical solvent composition, as evidenced by a variety of techniques commonly employed to characterize the aggregation of supramolecular polymers. These measurements show no structural differences for the equilibrated polymers up to the critical solvent composition of 15% ACN in water, the fraction of ACN below which clusters of solvent molecules do not yet occur.^31^,^32^ However, the HDX-MS experiments clearly reveal that increasing amounts of the cosolvent gradually increase the dynamic behavior of the polymers. The MD simulations show that the cosolvent has a destabilizing effect on the interactions between the monomers, and that its presence likely also influences the rate at which the molecules can leave and re-enter the polymers. Hence, these results demonstrate that the view we have of a supramolecular polymer is correlated to the time and length scales of the methods applied to investigate the assembly behavior. Our results illustrate that cosolvents that are not removed after the non-covalent synthesis of supramolecular polymers in water, have an important influence on the molecular dynamics even though no morphological changes can be observed.

The time-development of the polymerization and depolymerization of the supramolecular polymers was shown to be highly sensitive to the method of preparation. Fast polymerization was observed when molecularly dissolved monomers were injected from the good solvent into water; the self-assembly process occurred within minutes. However, during the depolymerization experiments that were initiated by mixing equilibrated polymers with dissolved monomers, the equilibration of the polymers took multiple hours. The required time for equilibration depends on the fraction of the good solvent. The longest equilibration time of the polymers was observed at the critical solvent composition. The large differences in the timescales detected in the polymerization and depolymerization experiments are proposed to be correlated with the main non-covalent interactions that are involved. In the polymerization experiments the hydrophobic effect is responsible for the observed fast kinetics. The different mixing protocol that was applied during the depolymerization experiments probably caused the formation of intermolecular hydrogen bonds to be the retarding factor in the equilibration of the polymers. The MD simulations show that this is a likely explanation because the good solvent was observed to both interfere with the hydrogen bonds, and to loosen the internal structure of the polymers.

Our results show the importance of kinetic measurements (1) to probe the influence of a cosolvent on the dynamics of supramolecular polymers in equilibrium, and (2) when monitoring the non-covalent synthesis of supramolecular aggregates in water. We expect that an increased understanding on the role of a cosolvent in water, in terms of molecular events, will contribute at a fundamental level to the development of multistep non-covalent synthesis protocols.^50^,^51^ Moreover, these insights will be crucial for all assembly studies of molecules in water that use a cosolvent in the assembly process.

## Conflicts of interest

There are no conflicts to declare.

## Supplementary Material

Supplementary information
