# Supplementary material for: Consequences of a cosolvent on the structure and molecular dynamics of supramolecular polymers in water
Source: Chem Sci. 2018 Jun 11;9(29):6199–209. doi: 10.1039/c8sc02257g (PMC6062890; doi:10.1039/c8sc02257g)
Supplement: Supplementary file 1 [file SC-009-C8SC02257G-s001.pdf]

Electronic Supplementary Material (ESI) for Chemical Science.
This journal is © The Royal Society of Chemistry 2018

Electronic Supplementary Information (ESI)

Consequences of a cosolvent on the structure and molecular dynamics of supramolecular polymers in water

René P.M. Lafleur,[a] Xianwen Lou,[a] Giovanni M. Pavan[b] Anja R.A. Palmans,[a] and E.W. Meijer*[a]

[a.] Institute for Complex Molecular Systems, Eindhoven University of Technology, P.O. Box 513, 5600 MB Eindhoven, The Netherlands. E-mail: e.w.meijer@tue.nl; Tel; +31 040 2473101

[b.] Department of Innovative Technologies, University of Applied Sciences and Arts of Southern Switserland, Galleria 2, Via Cantonale 2c, CH-6928 Manno, Switserland.

Instrumentation

UV-vis absorbance spectra were recorded on a Jasco V-650 UV-vis spectrometer equipped with a Jasco ETCT-762 temperature controller. Measurements were performed in quartz cuvettes with a path length of 1 mm, 1 cm or 5 cm and at a temperature of 20°C. First, a baseline of the corresponding solvent mixture was measured in the same cuvette as for the C_{12}BTA samples. All measurements were performed with a bandwidth of 1.0 nm, a scan speed of 100 nm/min and a data interval of 0.1 nm, spanning the UV-Vis range of 350 nm to 190 nm.

Light scattering measurements were performed on an ALV Compact Goniometer System (CGS-3) Multi-Detector (MD-4) equipped with an ALV-7004 Digital Multiple Tau Real Time Correlator and an Nd-YAG laser of 532 nm (with a power of 50 mW). Samples were measured in light scattering tubes made from glass with an outer diameter of 1 cm at 20 °C. These tubes were cleaned with acetone that was filtered using 0.2 µm PVDF (Supor membrane, PALL Corporation), and subsequently dried up-side-down. The water to prepare the C_{12}BTA samples was filtered with the same 0.2 µm PVDF filter, and the acetonitrile solvent was filtered using a 0.2 µm PTFE syringe filter (PTFE membrane, Whatman). Toluene was filtered with a 0.2 µm PTFE filter.

^{1}H NMR spectra were recorded on a 500 MHz Varian Unit Inova using pyrazine as internal standard at 500 µM, and deuterated solvents without TMS. The pyrazine used for these measurements was purchased from Sigma-Aldrich and added after equilibration of the water/ACN samples, about half an hour before starting the measurement. The chemical shift of pyrazine for the solvent mixtures was calibrated using VNMRJ.3.2.a software.

HDX-MS measurements were carried out using a Xevo™ G2 QTof mass spectrometer (Waters) using the settings described previously.[1]

Stopped-flow UV-Vis measurements were performed with a BioLogic stopped-flow setup consisting of a MOS-500 spectrometer with a Xe/Hg light source, an ALX 250 Arc Lamp Power Supply, SAS PMT-450 photomultiplier tube, an SFM-400/S stopped-flow module with an MPS-60 microprocessor unit and a Julabo F-12 temperature control system. BioKine 32 software was used to operate the stopped-flow setup. All measurements were performed at a wavelength of 229 nm, with a bandwidth of 1 nm and using a sampling time of 1 sec for short measurements (minutes) and 10 sec for long measurements (hours). The high voltage (HV) applied on the photomultiplier tube was set at a constant 340 V. Samples were mixed at different volume fractions and therefore flow rates, with the dead time of the machine below 15 ms. All measurements were performed using a BioLogic FC-15 cuvette with a path length of 1.5 mm, and at a temperature of 20 °C.

Samples for Cryo-TEM imaging were vitrified using a computer controlled vitrification robot (FEI Vitrobot™ Mark III, FEI Company). Quantifoil grids (R 2/2, Quantifoil Micro Tools GmbH) were surface plasma treated with a Cressington 208 carbon coater. Vitrified films were transferred into the vacuum of a Tecnai Sphera microscope with a Gatan 626 cryoholder. The microscope is equipped with an LaB_6 filament that was operated at 200 kV, and a bottom mounted 1024x1024 Gatan charged-coupled device (CCD) camera. For the kinetic studies as presented in Fig. 5 of the manuscript, an IKA T25 digital Ultra-Turrax was operated next to the Vitrobot.

Methods

BTA sample preparation. C_{12}BTA samples in water were prepared as described previously.[1] C_{12}BTA samples in acetonitrile were obtained by adding solvent to the solid. Prior to all measurements, except for the kinetic measurements, the equally concentrated C_{12}BTA solutions in water and acetonitrile were combined with micropipettes and equilibrated at room temperature overnight. The samples for the HDX-MS measurements were prepared by combining the solutions in water and acetonitrile using precise volume fractions. To this end, the volume fractions were converted into weight fractions using the density of the solvents. The weight fraction of acetonitrile that was added to the sample in water was accurately controlled with a microbalance. As an example, the procedure for 12% ACN is described. For an 850 uL sample, 12% ACN (v/v) will be 102 uL. This is 102 uL x 0.786 g/mL = 80.172 mg ACN. The contribution of water will be 748 uL x 0.998 g/mL = 746.504 mg. In weight percent the contribution of ACN will be 80.2 / 80.2 + 746.5 = 9.70%. Fresh C_{12}BTA solutions in water and ACN were prepared, both at a concentration of 0.75 mg/mL. 74.522 mg ACN stock was pipetted in a 1.5 mL glass vial on a balance with the cap on to prevent ACN evaporation. Subsequently, (74.522 mg x 90.30) / 9.70 = 693.90 mg of the C_{12}BTA in water at RT was added to it. The solvent mixtures containing D_2O that were used to dilute the samples just prior to the start of kinetic HDX-MS measurements were also prepared using a balance.

SLS and DLS. Scattering intensity and the autocorrelation of this intensity was detected over an angular range of 40° to 130° in steps of 5°, and averaged over 10 runs of 10 seconds, or 20 runs of 5 seconds per angle. Prior to the averaging, measurements showing obvious scattering from dust were removed using AfterALV (1.0d, Dullware) software. The Rayleigh ratios as a function of the angles were computed using the equation below with toluene as a reference.

$$R_\theta = \frac{I_{sample} - I_{solvent}}{I_{toluene}} \times R_{toluene} \times \frac{n^2_{solvent}}{n^2_{toluene}}$$

In this equation, *I* is the measured intensity of the sample, solvent mixture or toluene. *R* the Rayleigh ratio for toluene and *n* is the refractive index of the solvent or solvent mixture; these constants were used as previously reported.[2, 3] Second-order cumulant analysis of the intensity autocorrelation functions was performed in AfterALV software, and resulted in the characteristic decay rates. The predominant decay rates (γ) were plotted as a function of the scattering angle squared (q^2) in Fig. 2D. Linear regression yielded for 0% ACN $γ(q^2) = -509.4 + 5.1E6q^2$, for 10% ACN $γ(q^2) = -763.9 + 5.6E6q^2$, and for 20% ACN $γ(q^2) = 84.9 + 2.3E7q^2$. The adjusted R^2 of all of the fits is 0.99.

Vitrification for CryoTEM. Vitrified films containing C_{12}BTA in the presence of 15% ACN (equilibrated solution, Fig. 1C) or 10% ACN (mixed solutions, Fig. 5) were prepared in the 'Vitrobot' that was operated at 22°C, and at a relative humidity of 100%. In the preparation chamber of the 'Vitrobot', a 3 µl sample was applied on a Quantifoil grid which was surface plasma treated for 40 s at 5 mA just prior to use. Excess sample was removed by blotting using two filter papers for 3 s at –3 mm, and the thin film thus formed was plunged (acceleration about 3 g) into liquid ethane just above its freezing point. Vitrified films were transferred to the cryoholder and observed in the Tecnai Sphera microscope, at temperatures below -170 °C. Micrographs were recorded at low dose conditions, and at a magnification of 25000 with typical defocus settings of -10 µm (Fig. 1C) and -5 µm (Fig. 5).

Fitting of the kinetic UV-absorbance data. Sigmoidal fits were performed using the Boltzmann model in Origin 2015: y(x) = A2 + (A1-A2)/(1+exp((x-x0)/dx), and all adjusted $R^2 > 0.95$. Mono-exponential fits were performed using the Exponential model in Origin 2015: y(x) = y0 + Aexp(R0x), and all adjusted $R^2 > 0.94$. For both types of fits the t-90's were computed from the fitted curves. The first and last values of the fitted curve plots were used to obtain the UV-absorption values that correspond to 90% of the equilibrated value, and the corresponding time points were used to construct the graphs as displayed in Fig. 4B and Fig. 4C. Linear regression of the average lag times (Fig. 4B) yielded LagTime(perc) = 9.38 – 0.30perc, with adjusted $R^2 = 0.96$.

MD simulations. The atomistic model for the equilibrated water-soluble BTA fiber studied herein was taken from our previous work.[4,5] In the equilibrated fiber model, 10 mol% of the explicit TIP3P[6] water molecules present in the simulation box were replaced with acetonitrile (ACN) molecules. The force field parameters for ACN were taken from the literature.[7,8] The simulation work was conducted with the AMBER 12 software.[9] The atomistic model for the supramolecular polymer in explicit 90% water + 10% ACN cosolvent was first minimized and thermalized. Then, this was equilibrated for 250 ns of MD simulation in periodic boundary NPT conditions (constant N: number of atoms, P: pressure and T: temperature) at the experimental temperature of 20 °C and 1 atm of pressure. Owing to the anisotropic (1D) nature of the fiber models, anisotropic pressure scaling was adopted to allow the fibre to rearrange during the equilibration.[4,5] In the MD run, we used a time step of 2 fs, a 10 Å cutoff, the particle mesh Ewald[10] approach to treat the long-range electrostatic effects and the SHAKE algorithm to treat all bonds involving hydrogen atoms.[11] Several parameters were used to assess the equilibration of the simulated system in the atomistic MD regime: *i.e.*, solvent-accessible surface area (SASA), the fiber energy, root mean square deviation (RMSD), the average monomer incorporation energy (ΔH: solute-solute+solute-solvent) and the convergence of the calculated core-core and core-water radial distribution functions (*g(r)*). The last 100 ns of MD simulation

were considered as representative of the equilibrated BTA fiber in in 90% water + 10% ACN cosolvent, and used for structural and energetic analysis. The analysis of the hot spots in the fibers were conducted as described in our previous study.[12] The other structural analyses were conducted using the *ptraj* module of AMBER 12. Linear regression of the exchange hot spots (Fig. 7) yielded for 0% ACN SASA(ΔH) = -483.87ΔH + 2167.9, and for 10% ACN SASA(ΔH) = -476.28ΔH + 2106.9.

Supplementary Figures

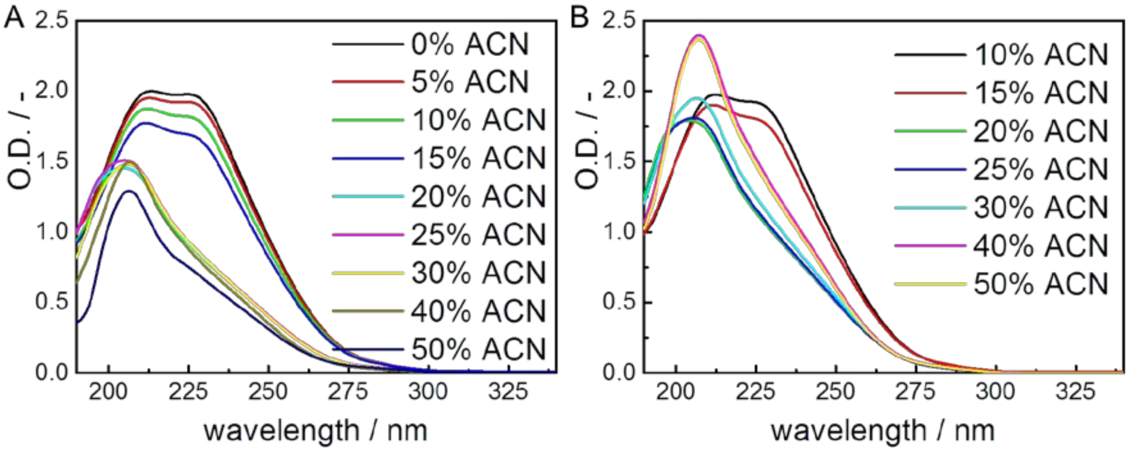

Fig. S1 A) UV-spectra that were obtained after adding ACN to C_{12}BTA polymers in water. B) UV-spectra that were obtained after injecting C_{12}BTA dissolved in pure ACN, into water.

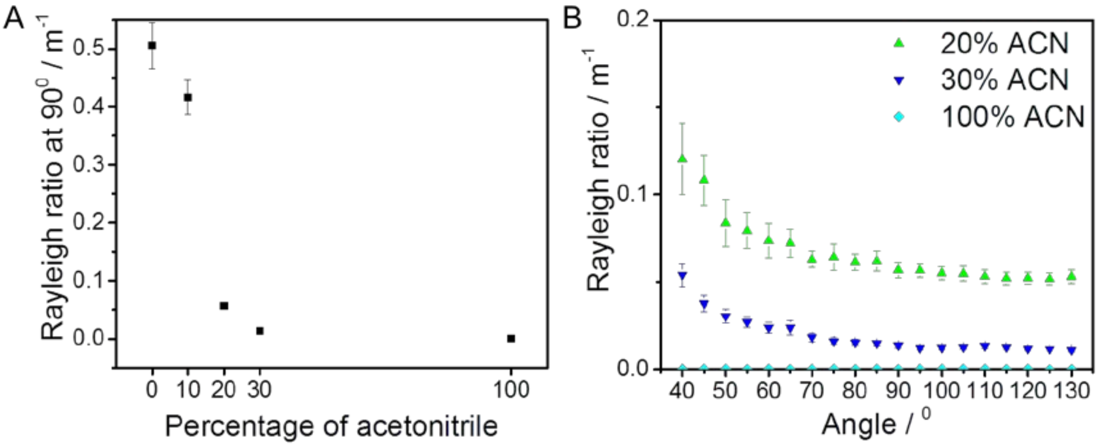

Fig. S2 Rayleigh ratio's, A) at 90° and as a function of the percentage of ACN, and B) at multiple angles for several percentages of acetonitrile.

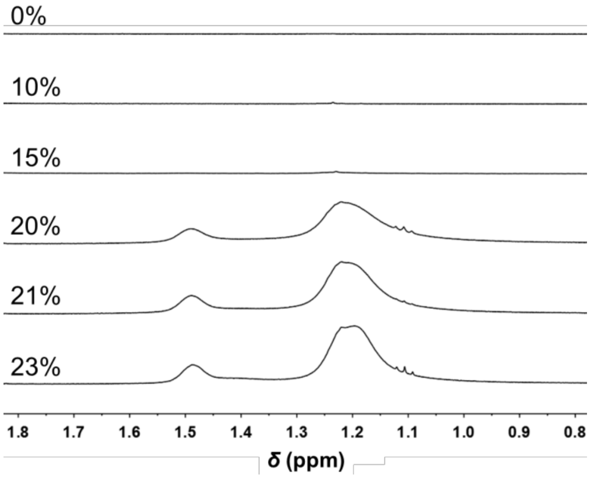

Fig. S3 Aliphatic region of ^{1}H-NMR spectra that were recorded at several ratio's of D_2O/CD_3CN. The measurements were performed at T = 20°C and at a C_{12}BTA concentration of 582 μM.

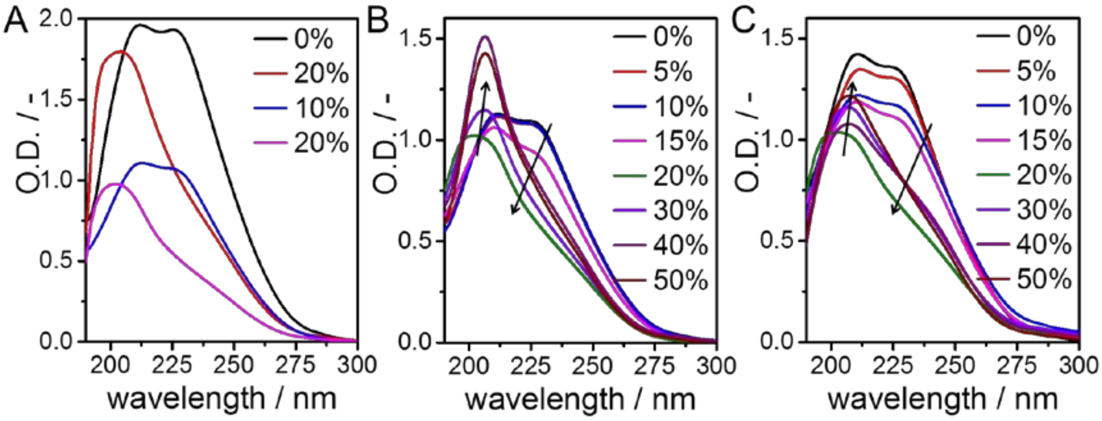

Fig. S4 UV-absorption spectra at various fractions of ACN in water. A) 582 μM samples were diluted 50-fold (black and red; 11.6 μM) or 100-fold (blue and purple; 5.8 μM) in solutions of equal solvent composition. Measurements were performed within 30 min after dilution (path length = 5 cm). B) 311 μM samples (path length = 1 mm), and C) 40 μM samples (path length = 1 cm), that were obtained by mixing equimolar solutions in water and ACN and storing the samples at room temperature overnight. All measurements were performed at 20 °C, arrows are added to guide the eye.

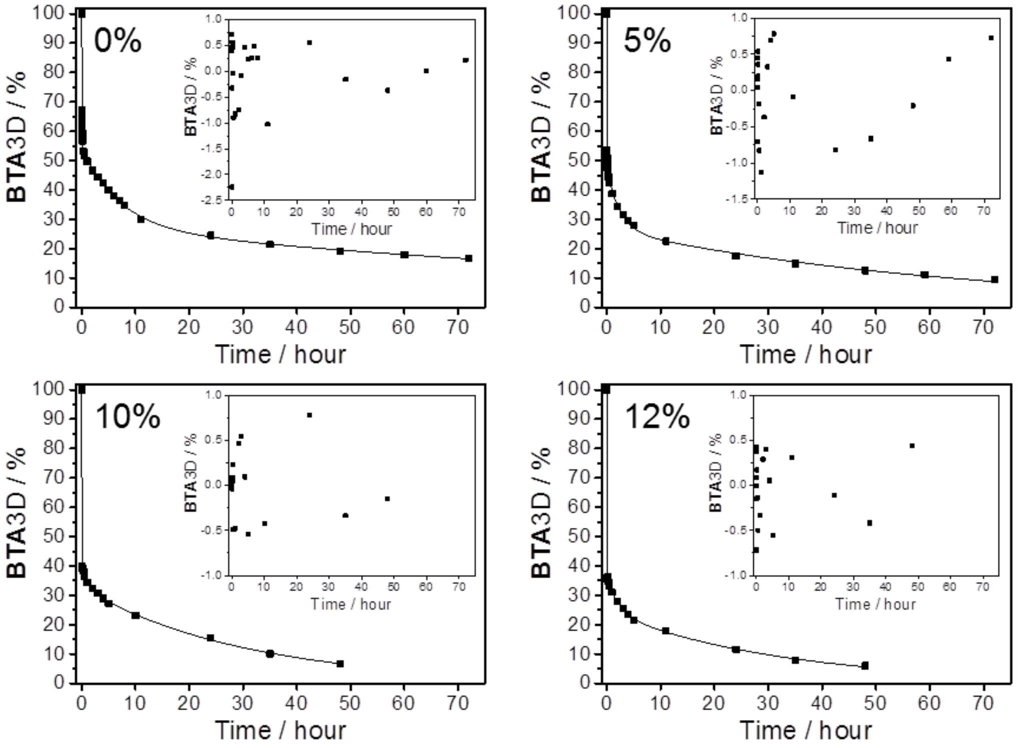

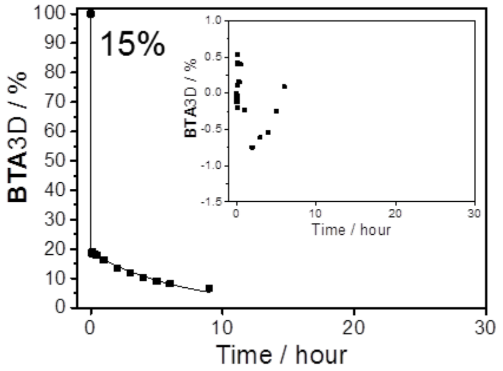

y(*t*)=**A** x exp(-*t* x **c**)+**B** x exp(-*t* x **d**)+ **E** x exp(-*t* x **f**)					
	0%	5%	10%	12%	15%
**A**	45.21	52.52	59.80	63.34	81.13
**c**	18.48	33.65	97.10	47E3	38E3
**B**	27.08	20.92	7.62	12.29	18.87
**d**	0.14	0.42	0.83	0.46	0.14
**E**	27.31	26.52	32.59	24.37	-
**f**	0.01	0.02	0.03	0.03	-

Fig. S5 Fitted curve plots for the HDX-MS data as presented in Fig. 2B, the insets display the corresponding residuals. The kinetic profiles < 15% ACN were fitted with a tri-exponential function. The fast decay in the presence of 15% ACN was fitted with two exponentials; all adjusted R^2 > 0.99.

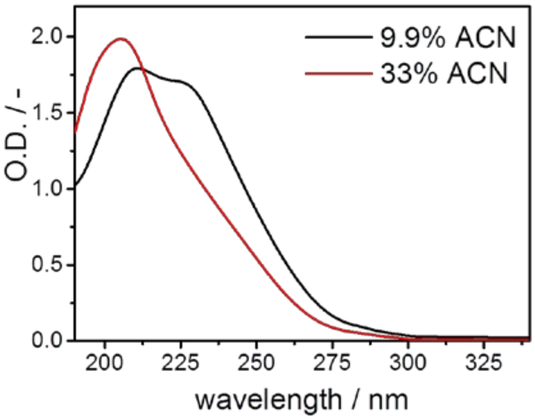

Fig. S6 UV spectra that were recorded 2.5 hours after mixing.

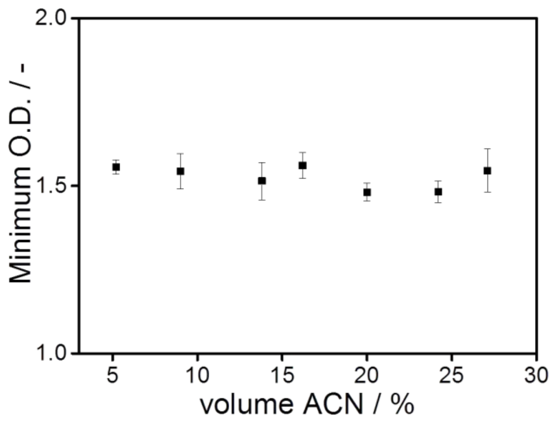

Fig. S7 Average minimum UV-absorption values at 229 nm, after the observed sudden drop in the UV-intensity. The values originate from the full kinetic profiles that were recorded (for example, see Fig. 4A), hence the squares are the average of three measurements. The error bars represent one standard deviation of uncertainty.

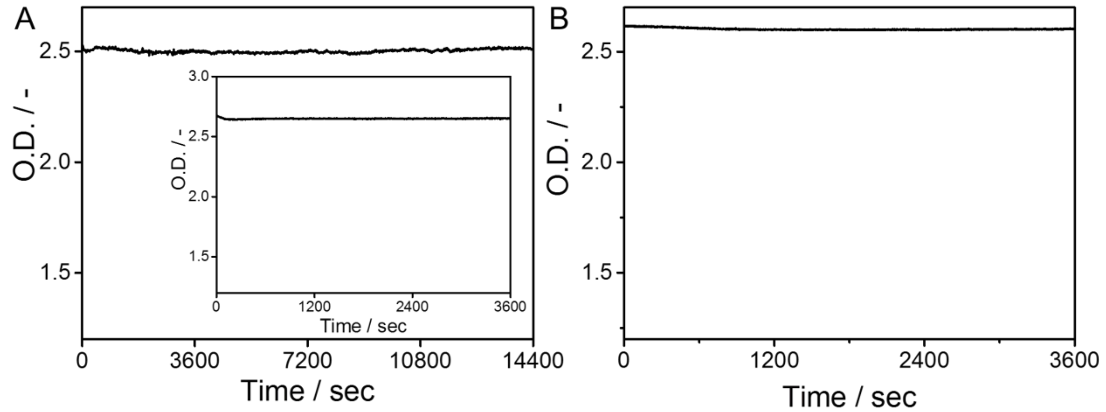

Fig. S8 A) Equilibrated supramolecular polymers in water were mixed equilibrated supramolecular polymers in water, or with monomers dissolved in ACN, resulting in 3% ACN in the final mixture (inset). B) Equilibrated supramolecular polymers in 95% H_2O + 5% ACN were mixed with equilibrated supramolecular polymers in 95% H_2O + 5% ACN.

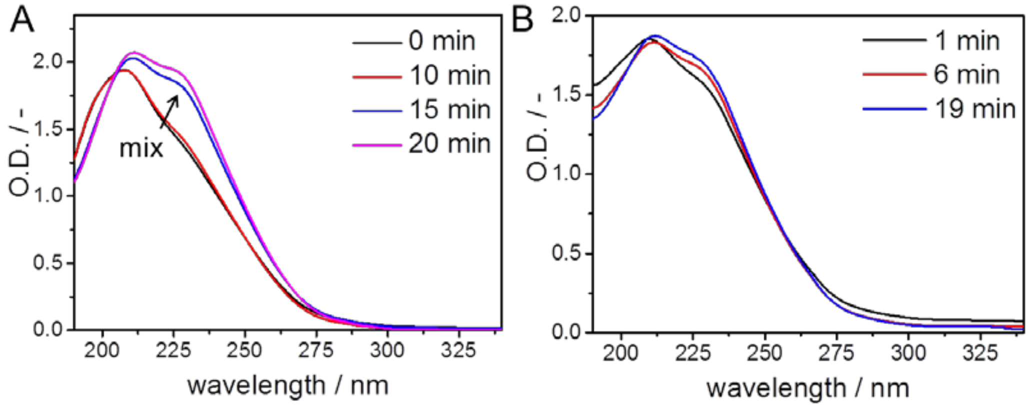

Fig. S9 Time-dependent UV-spectra at 14% ACN after; A) manual injection of a concentrated C_{12}BTA solution in ACN into water and inverting the cuvette three times after ten minutes for proper mixing, and B) collecting the mixed sample from the stopped flow machine into the cuvette.

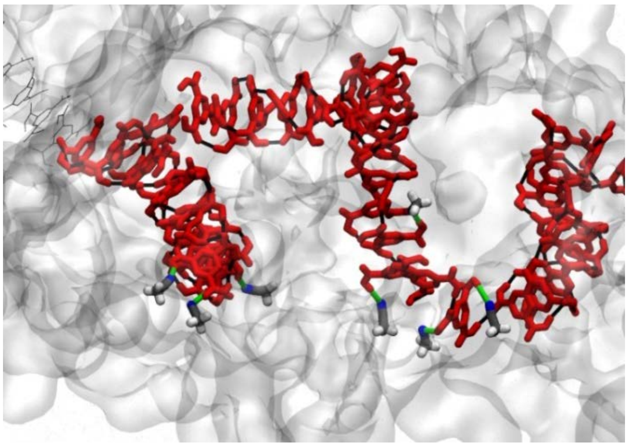

Fig. S10 Snapshot of the MD simulation showing the interaction of ACN molecules with the amides of the C_{12}BTA cores (shown in red) *via* hydrogen bonds (green).

References

1. X. Lou, R. P. M. Lafleur, C. M. A. Leenders, S. M. C. Schoenmakers, N. M. Matsumoto, M. B. Baker, J. L. J. van Dongen, A. R. A. Palmans and E. W. Meijer, *Nat. Commun.*, 2017, **8**, 15420.
2. H. Wu, *Chem. Phys.*, 2010, **367**, 44-47.
3. J. E. Bertie and Z. Lan, *J. Phys. Chem. B*, 1997, **101**, 4111-4119.
4. M. B. Baker, L. Albertazzi, I. K. Voets, C. M. A. Leenders, A. R. A. Palmans, G. M. Pavan and E. W. Meijer, *Nat. Commun.*, 2015, **6**, 6234.
5. M. Garzoni, M. B. Baker, C. M. A. Leenders, I. K. Voets, L. Albertazzi, A. R. A. Palmans, E. W. Meijer and G. M. Pavan, *J. Am. Chem. Soc.*, 2016, **138**, 13985.
6. W. L. Jorgensen, J. Chandrasekhar, J. D. Madura, R. W. Impey and M. L. Klein, *J. Chem. Phys.* 1983, **79**, 926.
7. F. Bardak, D. Xiao, L. G. Hines Jr., P. Son, R. A. Bartsch, E. L. Quitevis, P. Yang and G. A. Voth, *Chem. Phys. Chem.* 2012, **13**, 1687.
8. A. M. Nikitin, A. P. Lyubartsev, *J. Comput. Chem.* 2007, **28**, 2020.
9. D. A. Case, *et al., AMBER 12* University of California, 2012.
10. T. D. Darden, D. York and L. Pedersen, *J. Chem. Phys.* 1993, **98**, 10089.
11. V. Kräutler, W. F. van Gunsteren and P. H. Hunenberger, *J. Comput. Chem.* 2001, **22**, 5385.
12. D. Bochicchio, M. Salvalaglio and G. M. Pavan, *Nat. Commun.*, 2017, **8**, 147.
